# Supplementary material for: Epicardial adipose tissue and carotid artery disease: Protocol for systematic review and meta-analysis
Source: Medicine (Baltimore). 2018 Apr 27;97(17):e0273. doi: 10.1097/MD.0000000000010273 (PMC5944522; doi:10.1097/MD.0000000000010273)
Supplement: Supplemental Digital Content [file medi-97-e0273-s001.pdf]

PubMed

Patient

#1 " **Epicardial Adipose Tissue and Carotid Artery Disease** "[Mesh]

#2 Epicardial Adipose Tissue and Carotid Artery Disease *[Title/Abstract]

EMBASE.com

Patient

Patient

#1 " Epicardial Adipose Tissue and Carotid Artery Disease "[Mesh]

#2 Epicardial Adipose Tissue and Carotid Artery Disease *[Title/Abstract]

Cochrane Library

#1 " Epicardial Adipose Tissue and Carotid Artery Disease "[Mesh]

#2 Epicardial Adipose Tissue and Carotid Artery Disease *[Title/Abstract]

Embase

#1 " Epicardial Adipose Tissue and Carotid Artery Disease "[Mesh]

#2 Epicardial Adipose Tissue and Carotid Artery Disease *[Title/Abstract]

Google scholar

#1 " Epicardial Adipose Tissue and Carotid Artery Disease
